# Address Delivered before the Cincinnati Association of Dental Surgeons

**Published:** 1844-06

**Authors:** M. Rogers

**Affiliations:** President of the Society.


### ARTICLE VI.

*Address delivered before the Cincinnati Association of Dental Surgeons*, February 2d, 1844. By M. Rogers, M. D., President of the Society.

Dentistry may be considered as a science, and as an art.

As a science it teaches—the structure and uses of the human teeth, and the parts associated with them in the functions of mastication and speech.

It treats of the vicissitudes incident to the teeth in the progress of life, their natural wear and tear, the diseases peculiar to the teeth themselves, such diseases of the gums and other parts of the mouth as effect their beauty, usefulness and durability, of the direct and indirect effect of the diseases of other parts of the body upon the teeth.

As an art it teaches the use and application of the means necessary to promote the healthful growth and regularity of the teeth, the cure and precaution necessary to preserve their health and beauty. The treatment necessary to arrest decay when it attacks their substance, and for the cure of those diseases which waste and destroy those parts upon which they depend. It also teaches a thorough knowledge of those delicate chemical and mechanic arts necessary to enable the dentist to substitute and adapt with the most minute exactness, artificial structures to the mouth, to represent in perfection the living beauty of teeth that have been lost.

This definition of dentistry will suggest some idea of the *importance* of our profession and the deep interest that society have at stake in the character and ability of those who practise it.

I propose to devote this paper to the consideration:

Of the importance of the profession to which we belong.

The moral obligations resting upon those who practice it: and, to

Offer some hints upon the rules and principles that must govern the dentist to enable him to gain the confidence and patronage of an enlightened community.

To get a proper idea of the importance of our profession. It is well to inquire who are the subjects of it? It is not with us as with the profession of medicine, where the lame, the blind, the halt, the poor and the distressed in life, get the largest share of professional labor. Our principal business is performed for the most valued, the most useful, and the most beloved members of society.

The importance of our profession is shown by the great value of the teeth and how indispensable they are to comfort and happiness. The proper mastication of the food is so important a link in the process of digestion, that if this, the first preparation of the aliment, is improperly performed, dyspepsia is very liable to be induced, a disease most difficult to cure after the teeth have been lost. I need not dwell upon the numberless concomitant evils which may owe their origin to imperfect mastication.

We are called next to contemplate the importance of the teeth as they stand connected with the grand prerogative of man—the GIFT OF SPEECH. The teeth are absolutely necessary to perfect articulation, and to the modulations of the voice. Their preservation is therefore essential to all persons who do not exclude themselves from society.

For what can be more disagreeable than the mumbling conversation of those who have lost their teeth.

To all public speakers, teeth real or artificial are a "*sine qua non*," without them the graces of eloquence are lost.

It would be well if all persons could be impressed with these facts before it is too late, as it would prove one of the strongest inducements to bestow upon the teeth the care necessary to their preservation.

We come next to contemplate the teeth as an element of beauty. And here they must ever stand pre-eminent.

No regularity of features, brilliancy of complexion, sparkling of eyes, or braids of jetty hair, can render a lady beautiful, who, when she opens her lips, discloses a set of teeth irregular or discolored.

A mouth large and ill-formed, will pass very agreeably if it contains teeth beautifully white and even. In short, good teeth, well kept, will prove a redeeming feature to the face, even if the others be indifferent.

On the other hand, the effects of decay, the presence of tartar, the blackened fangs, the taint of the breath, always accompanying such a state of the mouth, is both loathsome and disgusting. A face ever so finely formed, with the elements of beauty interwoven in every lineament, is absolutely painful to look upon, if it has in the foreground, a mutilated set of teeth.

A voice set by nature in the sweetest tones of melody, as if intended only to put forth those sounds, and utter those sentiments that delight the ear, and captivate the heart, how uninteresting and painful it becomes by the lisping and halting occasioned by the loss of teeth.

The value set upon these beautiful organs by that sex, to whom, man with all his boasted independence, looks for the centre and foundation of his earthly happiness, for whose smiles we labour, and for whose favor we repine, I say the value which they set upon these beautiful organs must always form a crowning argument for the importance of that *art*, which has power to preserve from decay, and ensure their beauty and utility through life.

That well regulated dentistry has this power, we all believe, and let us all here stop and resolve, that *it shall always have this power* while confided to us. It will be well for society if it does, and a fearful responsibility rests upon us if it does not. My friends, shall this beautiful, smiling, hoping young daughter, doted upon by her father, worshipped and idolized by her mother, and beloved by every body, shall she be mutilated in our hands; a principal element in her beauty taken away, trifled with, and destroyed by her dentist? Let him who would do such a thing, be execrated and pointed at till he find his proper place in or out of society. Let not a liberal profession be disgraced by the man, who, for want of skill, or through negligence would, or could perpetrate such an evil upon a confiding fellow-being.

The enlightened part of society understand well, the importance of dentistry, and they are willing to pay us well if we will do our duty faithfully, and they may also be willing to pay *him well* in coin that he may not relish, who, through ignorance or neglect of duty, betrays their confidence. I say again, let us resolve to be faithful.

The great delicacy of the organs which we are called to treat, their exquisite sensibility, and the great influence which their diseased condition exerts upon the rest of the system, shows the importance of our profession; and it gives, too, some little hint of the circle of science which an accomplished dentist must attain, to make him master of, or qualify him for its successful practice.

It will be well for society when the practitioner in this branch of surgery, shall be compelled by law, and what is still better, by public sentiment, to be a graduate of a medical college before he is allowed to assume the responsibilities of our noble profession. We are not consulted merely to prepare a cosmetic, a wash, or a powder, to adorn and beautify the teeth, or give a grateful perfume to the breath: our duties lie much higher than this.

We are often called upon to determine whether these beloved members of society shall have teeth of their own, or be subject to all the pain and mortification which attends the loss of one of the chief elements of beauty and comfort for the rest of their lives, and be liable to be in the power of the very man whose ignorance and mismanagement has thus mutilated their persons.

Our profession being one of the liberal and scientific, places the practitioner in a conspicuous situation in society, and lays him under strong moral obligations to sustain the character and dignity awarded by society, to members of the learned professions. We are morally bound as individuals, and as brethren having a common interest to maintain, and mutual duties to perform to each other, to cultivate the kindest fraternal feelings. Let our social intercourse be regulated by that gentlemanly courtesy characteristic of an elevated mind, actuated by generous feelings and a conscientious regard to duty. Without maintaining such an intercourse, we are in danger of lowering our profession in the estimation of society, and gaining for ourselves little else beside ridicule and contempt.

The necessity of strictly adhering to these rules, when our patients or others are disposed to question the character and ability of a brother, or even when they have a just cause of complaint, is very manifest.

From what cause it arises, I do not pretend to say, but we are often called upon to listen to long complaints against the practice and management of a professional brother. We are thus placed in a very critical and a very trying situation. In these circumstances, my friends, I know of but one rule that a man can pursue and keep in the path of duty, viz. "to do unto others as you would wish them to do unto you." It is the only rule that applies in the case, and it is a safe one. I hope there is not an individual in the sound of my voice that will ever do otherwise. In such a case, we are bound by every consideration to conciliate feelings, to explain mistakes as far as we are able, and do every thing that an honest man can do to protect a brother from unmerited injury. I say *unmerited,* for I have rarely met with a case of the kind where the complainer did not do more or less injustice by suspicions or misapprehensions of the case, or the motive of the practitioner.

Community is full of those who seem to delight in fault-finding, and there are others who are honest, perhaps, in their intentions, but who never seem to have a correct idea of anything, and they are the very ones who talk the most, and are always the most confident.

Now, my brethren, we shall all have to take our share in these things; there is not one present, whose reputation for the time being, may not be entirely in the hands of a brother under such circumstances.

If we join with such a person, and allow a feeling of envy or jealousy to govern or influence us, we may, indeed, injure a rival, or gratify a private pique, but do we look a little further and see that upon cool reflection, the very person we encouraged will despise us, while on the other hand, if we spoke of our brother's faults with gentlemanly caution or regret, set the good points of his character in view, and in a word, showed sorrow and regret at the thought of our profession suffering in the person of any of its members; I put it to any one, would not such a course command a respect, would it not indicate a christian spirit, and would not even the detractor himself feel more safe in the hands of such a man? "Virtue, indeed, carries with it its own reward."

Then let us keep in mind the high moral obligations that rest upon us, as practitioners, as men, and as christians; do every thing in our power to elevate the character of our brethren, individually, and by that means lay deeper the basis of our own.

The generous confidence which our patients manifest, by placing interests so dear to them, in our power, increases our moral responsibility; it is one from which we cannot escape. Then let us make ourselves worthy of such confidence by the ability and moral integrity of men, not only skilled in the mysteries of their profession, but whose hearts and principles of action are formed and fashioned by our Saviour's golden rule.

Shall we destroy our neighbor's teeth, endanger his health, and take his money too? All this is easily done, aye, often done by a careless performance of our duty. We have many temptations to contend with just now, and one which besets us almost every day, is the very honest one perhaps on the part of our employers, of wishing to get their work done cheap.

People have not yet learned that cheap dentistry is as plenty as any other cheap thing, and worth about as much in comparison. But my friends, let me warn you against temptations to sell cheap dentistry. By dealing honestly and faithfully with those who do not suspect that cheap dentistry is among the dearest things on earth, we may give them advice most valuable, which will prevent them from again annoying us by trying to beat down our prices. People are not always to blame for wishing to get their teeth operated on for a reasonable price, or for wishing to know your price beforehand, because our profession has been disgraced and outraged by those who had no other rule to regulate their charges, than to be sure and get all the money they can from every one that employs them.

It is our duty to disabuse ourselves and our profession from this charge, and impress upon the mind of every one, that we have a fixed and unvarying standard of prices, and convince them that our standard is the reasonable and strictly just one, to charge just as much as we fairly ought to have and no more, and of charging that uniformly, and also of rendering our services honestly and faithfully at all times. I say let us *convince* every one that we are governed strictly by these rules, and we shall soon find that we are saved this most trying and vexatious annoyance of being jewed in our prices. I am greatly mistaken if a truly capable man will not find a reasonable support in this community; he has only to make himself *capable* and *worthy* of support to get it. That man who will lower his prices to suit the tastes or prejudices of his patients, will always be sure of getting two things, one of which will be, the distrust of his patients, and the other necessarily must be, a practice not worth having.

There are two things positively and unconditionally necessary to success in business, to wit:—capability and moral honesty. Without these a man could as well navigate a ship without a compass or a rudder, as to expect continued success in an enlightened community. To be honest we must have a correct and an unvarying rule of action, and what is of equal importance to our success, we must act so as to let the public know we have it. Little tricks and deceptions covertly practiced, may do in some kinds of business, but it will not do in ours. We are public characters, and as such our professional conduct must be strictly honest, open and decided at all times. Our responsibilities are so great, that society will not let our acts pass without strict scrutiny.

When the fond parent places his child under our treatment, he pays the highest compliment in his power, to our professional ability. What does he merit in return? Is it not the best skill and service of which the profession is capable? Do we always give it? Have we it always to give? If not are we morally honest? Are we capable?

These questions come home to heart and conscience, and they ought ever to be there while we practice dentistry.

I have been sixteen years a practitioner in Cincinnati; I have always found this community generous and confiding, and I always believed the little success that I have met with, was owing more to an open, candid course of conduct, than to any other cause; I never fail to recommend the same to others, on all suitable occasions.

With regard to capability, I have one word to say, and the first is, *look to your books.* They contain the principles of science upon which dentistry is founded. But do not think, after you have read Bell, Fitch, Koecker, Maury, &c., all of them good and practical writers on our noble art, that you have all that the books contain on the subject of dentistry, or enough to make you capable, in any reasonable sense of the term. They have only the upper works; the foundation lies entirely below them.

We need a knowledge of chemistry, to teach us the laws that direct even our mechanical operations, much more the great laws that govern the physical properties of these beautiful, these sensitive organs, so especially entrusted to our care. We need anatomy, to teach us their structure, and the structure of those parts of the body upon which they depend. We need physiology, to teach us the laws of life that govern, direct, and *make* these delicate sensibitities. We need pathology, to teach the phenomena of the diseases of the organs we are called to treat. We need a most thorough knowledge of the principles and practice of surgery, as the finishing and crowning part of our professional character.

My friends, society expects all this of the man who assumes the character and pretensions of a DENTAL SURGEON. They expect him, also, to be a gentleman and an honest man.

In conclusion, for the student to be fitted for the practice of dentistry, it is essential that he should cultivate not only his intellectual and mechanical powers, but he must cultivate the heart and conscience, till he is actuated by an elevated and refined standard of moral principle and feeling, that shall govern all his intercourse with his fellow men.

United with industry and prudence, such a standard, faithfully followed, will secure ample pecuniary support, gain the affectionate confidence of society, and make him an ornament to his profession.

